# The Impact of COVID-19 Pandemics on the Development of Health Risk Communication: Challenges and Opportunities

**DOI:** 10.3390/ijerph20010645

**Published:** 2022-12-30

**Authors:** Antonio Valenti, Marco Mirabile, Erika Cannone, Fabio Boccuni, Pierluca Dionisi, Grazia Fortuna, Diana Gagliardi, Romina Vizzaccaro, Sergio Iavicoli

**Affiliations:** 1 Department of Occupational and Environmental Medicine, Epidemiology and Hygiene, Italian Workers’ Compensation Authority (INAIL), Via Fontana Candida 1, Monte Porzio Catone, 00078 Rome, Italy; 2 International Commission on Occupational Health (ICOH), Via Fontana Candida 1, Monte Porzio Catone, 00078 Rome, Italy; 3 Directorate-General for Communication and European and International Relations, Ministry of Health, Lungotevere Ripa 1, 00153 Rome, Italy

**Keywords:** COVID-19, communication strategies, infodemic, Italy, risk communication

## Abstract

Starting from an analysis of communication in Italy during the COVID-19 emergency period (February–June 2020), this paper provides an overview of the main challenges and opportunities for communication during pandemics. The purpose of this study is to perform a literature review contributing to the identification of practical recommendations for the improvement of current risk communication strategies. Given the variety of the parties involved in communication and the peculiarity of the theme, an integrated analysis approach was adopted, based on the connections between institutional, scientific and mass communication. On one hand, the “emotional” character of Italian institutional communication aimed at promoting solidarity and unity among citizens. On the other hand, scientific communication played a key role both as a technical and scientific consultation for the policymaker, and as a guide for mass communication. Nevertheless, a lack of awareness emerged from the institutional and scientific side of the importance of an interface between science and effective, transparent policy. It thus becomes necessary to develop new and effective communication strategies aimed at facing uncertainties and the challenges of risk communication in epidemics and pandemics. Such strategies should consider interaction between public health, human and social sciences, political science, law, ethics, communication and media studies, as each of these areas may give an important contribution to the understanding of the context in which communication occurs.

## 1. Introduction

In the last decades, the World Health Organization (WHO) has recognized the importance of risk communication, defined as the process of the real-time exchange of information, advice and opinions between experts or officials and people, as a key factor for public health emergencies preparedness and response like epidemiological analyses, and the adoption of pharmacological and non-pharmacological measures. [1,2,3]. 

For this reason, accurate information provided in timely fashion, and in languages and channels that people understand and use, may improve the awareness and engagement of communities, promote the acceptability of government measures, enable individuals to gather information on risks and to change their behaviors to minimize or prevent risks [4,5].

The development of pharmacological and non-pharmacological measures, and especially vaccines, has proved effective over time because it is grounded on scientific evidence and consolidated methodologies used since the seventeenth century [6,7]. The COVID-19 pandemic, instead, revealed the weaknesses of risk communication in a context characterized by uncertainty and insufficient medical and scientific knowledge of SARS-CoV-2. Such circumstance induced confusion in the public and represented a challenge for evidence-based decision making in Italy [8]. In pandemics, emergency crosses health protection, hitting the crucial relationship between individual health and public health. 

On 30 January 2020, the WHO declared the outbreak of COVID-19 to constitute a Public Health Emergency of International Concern (PHEIC) [9]. From that moment and for the following months, information searching increased, regarding different aspects: the number of cases, hospitalizations, and fatalities; prevention and distancing measures; restrictions, etc. [10,11].

**Figure 1:**
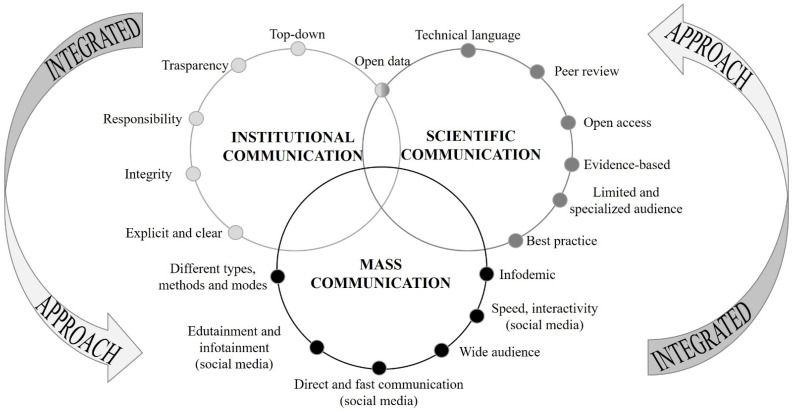
Intersection of institutional, scientific and mass communication.

Adding on the complexity of the object of communication itself, i.e., the pandemic, other difficulties arose from the communication modalities, characterized by the intersection of three types of communication (Figure 1): (i) scientific communication by experts, an essential starting point for the understanding of the pandemic and its development; (ii) institutional communication, dealing with strategies adopted by the national institutions; and (iii) mass communication, through which both scientific and institutional communication reached the public, by simplifying or amplifying the message.

The widespread use of information and communication technologies (ICTs) played a crucial role in disseminating information and enabling a larger number of people to access information. However, excessive amounts of information represented a threat to public health, and also because of the spreading of inaccurate and altered information (infodemic), affecting people’s behavior and, consequently, public health [5]. 

The Internet and social media are fertile land for alternative facts and fake news, because there is no coherent fact checking system on the Internet. Information and fake news fuse together, and it is up to the user to filter a large amount of information according to their own capabilities, which are often influenced by cognitive biases (confirmation bias, cherry picking), the lack of willingness to check facts, inadequate digital literacy, and poor health literacy [12].

A study developed by the Italian foundation *Fondazione Bruno Kessler* (FBK), analyzing the reliability of information sources from 22 January to 10 March 2020 with an infodemic risk index (IRI), showed that the infodemic risk was higher during the first period, with the spread of a large proportion of potentially fake news. When the infection started spreading in many countries, a higher amount of information from reliable sources was shared, thus the infodemic risk decreased. This was particularly evident in Italy, where infodemic risk decreased dramatically in such a short period, falling from 0.061 to 0.011. In the same period, the percentage of online messages about COVID-19 containing reliable information increased from 73.61% to 80.98% [13].

More specifically, in Italy, 50 million people (99.4% of the total adult population) searched for information on the ongoing health emergency. This had never happened before. The Internet was the favored place where misinformation and fake news originated and spread from: 29 million Italians declared having read news on the web that later turned out to be false or incorrect [10].

During the period January–March 2020, a continuous growth is observed in the proportion occupied by COVID-19 news in both information and misinformation sources. Posts or tweets on the novel coronavirus increased by 37% for information sources, while a 28% increase is observed for misinformation sources. With the passing of time, the number of pandemic-related news items started decreasing in online misinformation sources; a decrease that is less marked than the one observed for information sources [14].

Starting from an analysis of communication in Italy during the COVID-19 emergency period (February–June 2020), the present study aims to stimulate discussion on the importance of communication in public health crises, and, consequently, seeks to contribute to the identification of practical recommendations for the improvement of current risk communication strategies.

## 2. Methods

This study is a general literature review that provides a comprehensive, critical and objective analysis of the current knowledge about risk communication in health emergency crises [15,16].

Analysis takes into consideration the period from February to June 2020, when data and information on the COVID-19 emergency began to prevail in institutional and scientific communication in Italy, immediately after the news of the first two confirmed COVID-19 cases in the country (30 January 2020). That period was the peak of the health emergency. It was characterized by a large spread of information, which were not always reliable, giving rise to the phenomenon known as infodemic.

We searched for potentially eligible studies in grey and peer-reviewed literature databases, Internet search engines, organizational websites and major electronic academic databases. The search strategy was built into PubMed syntax using the following key words (both as MeSH terms or quoted sentences and loose words in the title and abstract field): risk communication, COVID-19, pandemic, coronavirus, SARS-CoV-2, scientific communication, institutional communication, infodemic, risk perception, social media, communication strategies, Italy. 

The analysis also takes into account technical documents and reports developed by the major institutional websites: (i) Italian National Institute of Health—ISS: https://www.iss.it/rapporti-covid-19 (accessed on 26 May 2022); (ii) Italian Ministry of Health: https://www.salute.gov.it/portale/nuovocoronavirus/homeNuovoCoronavirus.jsp (accessed on 26 May 2022); (iii) Italian Workers’ Compensation Authority—INAIL: https://www.inail.it/cs/internet/comunicazione/pubblicazioni/catalogo-generale.html (accessed on 26 May 2022). 

We also analyzed the reports of the Technical-Scientific Committee (CTS), created in February 2020 at the Civil Protection Department and responsible for advising and supporting the coordination activities to overcome the epidemiological emergency of COVID-19.

The critical appraisal of all the documents retrieved through our search, allowed developing the integrated analysis approach, which highlighted the connections between institutional, scientific and mass communication and, allowed us to identify the main challenges and opportunities for communication during pandemics, and, ultimately, to offer practical recommendations for the improvement of current communication strategies.

## 3. Institutional Communication during COVID-19

Many studies in political and administrative science have shown how institutions change their communication strategies in crises, to guarantee transparent procedures and decisions, and to alleviate anxiety caused by uncertainty and the lack of information [17,18,19,20].

In particular, the multi-dimensional crisis caused by the COVID-19 pandemic has contributed to a growing role of institutional communication in influencing the public’s perceptions and behavior, scientific research, vaccine development, health system and economic measures for pandemic containment [21]. This produced the positive effect known as “rally-around-the-flag”; namely, a short-term increase in the popularity of political leaders and governments in times of crises [22,23].

Institutional communication, intended as communication occurring between public administration and population, played a fundamental role in informing the citizens about behaviors and rules to follow to contain the spread of the infection [11].

During the first month of the pandemic crisis, Italy was one of the most exposed countries to health risks, and thus it needed to prepare a highly responsive and effective risk communication strategy [24].

In this sense, Italy was considered as a prime example for pandemic crisis management; also, because of the choice of adopting “emotional” communication through the use of simple language, which conveyed a sense of unity, belonging, communion, and solidarity [25,26] to reassure the public while the crisis was reaching its peak, with increased fatalities and the overload of hospitals with patients [27]. 

Communications from the Italian Government about measures adopted to contain the pandemic and the related social and economic impacts were drawn in the Decrees of the Italian President of the Council of Ministers—DPCM. Apart from that, the core of institutional communication was a “quantitative” element, consisting of daily reports on the numbers of new cases, hospitalizations, ICU treatments, fatalities, recovered patients, etc.

Such reports were delivered by institutions and specific bodies such as the ISS, CTS, research institutes, universities, etc., creating interconnections between institutional and scientific communication [11].

Daily press conferences were activated by the Civil Protection Department, with the participation of experts from the CTS. Such press conferences ensured continuous connection with the population during the earliest phase and the lockdown. Press conferences of the Ministry of Health and ISS and their public notices still continue to be a relevance and transparency tool. Dashboards on epidemiological data, ICU beds, daily tests and vaccines represent a constant and direct system for the accountability of regulation, monitoring and contrasting actions.

Institutional communication employed traditional storytelling techniques. Drawings were used to show, in a simplified way, the restrictions that the population would have to observe: when and how to use face masks, when leaving home was or was not permitted, distancing measures, self-declarations for leaving home, closures and reopening. Visual storytelling proved useful when the message to be conveyed concerned scientific data, hard to understand for many [28].

Institutional communication was also affected by some limits. The continuous development of the pandemic led to a monopolization of the themes, and this produced an effect of redundancy. Furthermore, institutional communication expanded across three levels (national, regional and municipal), and this produced some difficulties in the harmonization of communication itself.

Institutional communication was linked to mass communication. Two aspects should be considered. Firstly, institutions used mass media and social media to reach the public. Secondly, mass media and social media were a vehicle for the spread of distorted information, not in line with the official, institutional information sources [11].

Because of the spread of a large amount of information, not always reliable, all institutions involved, starting from the Ministry of Health, established dedicated communication channels, enhanced their use of social media, and put in place communication campaigns; they also focused on specific areas of interest. 

For example, the social media pages of the Italian Ministry of Health increased from 110,000 (September 2019) to 3 million followers, as of June 2022 [29]. 

During the first two months of the pandemic phase, 301 posts were published on the Italian Ministry of Health Facebook page, 94% regarding COVID-19. Messages countering fake news covered 7.1% of the institutional flow. The number of views increased significantly, reaching an eight to ten times higher range than before the pandemic, and it is still very high. 

As one of the earliest measures taken in contrast to the infodemic, the Italian Ministry of Health started a partnership with the major search engines to reorganize search results appearing on the page [30].

Furthermore, since the initial phase of the pandemic, a fake news section was included in the Ministry website, both in Italian and English, to monitor the most popular fake news and compare them with “genuine” news. 

Another institution, the Italian Communications Regulatory Authority (AGCOM), adopted similar actions. Among their monitoring activities on on-line platforms to contrast misinformation on COVID-19, AGCOM included a project proposed by Facebook based on a new fact-checking service for COVID-19 news and information, available on WhatsApp [31].

The Italian Government created campaigns involving famous people and digital influencers, and using specific hashtags (e.g., #iorestoacasa). These posts were enriched with emoticons, infographics and social cards, frequently integrating the word falso (false) or “fake news” in visuals, and linking to the COVID-19 section in the ministerial website [30]. 

This was described as a hybridization process between a “one to many” model, typical of mass communication, and “one to one”, “many to many”, “many to one” models, characteristic of the platform society [32].

In addition, participation of institutional testimonials at radio and TV programs and the involvement of the spokesperson of Technical-Scientific Committee for COVID-19 contributed to keeping constant communication in line with contemporary communication.

## 4. Scientific Communication during COVID-19

During the pandemic, scientific communication had a key role in producing evidence-based knowledge and, consequently, in providing technical and scientific advice to the policymaker and guiding mass communication. Several aspects affected and complicated the development of communication strategies, especially during the initial phases. Such aspects included: (i) the lack of consolidated scientific knowledge on the epidemiological features of the novel virus; (ii) the consequent lack of scientific certainties about the most effective and appropriate containment measures; (iii) difficulties in containing infodemics, due to the gradual development of scientific knowledge and to the large dissemination capacity of pseudo-scientific news through social media; and (iv) difficulties in risk evaluation, to avoid underestimations and unnecessary alarms [33,34,35]. During the early phase of the pandemic, mathematical models of the epidemic curve, epidemiological parameter estimates, simulation of the effects of non-pharmaceutical interventions, and predictions of the impact of COVID-19 on hospitalization days (also in intensive care units) and fatalities were divulged [36].

Such focus on an unprecedented public health event led to a phenomenal growth of scientific production, with a deepened interest for domains that are not strictly related to the bio-medical field. An example is the development of a high number of peer-reviewed publications in a short time span [37].

We conducted an analysis on PubMed using the keywords “COVID-19” and “SARS-CoV-2”. As of May 2022, we found 266,000 peer reviewed publications, 93,000 of which were published in 2020. To better understand such information, it may be useful to compare this with the total number of scientific publications on malaria, one of the most well-known transmissible diseases that still has an impact on public health. The number of scientific publications on malaria is 2.5 times smaller.

Furthermore, peer review processes for COVID-19-related publications proceeded faster than usual. Pre-print platforms such as medRxiv were also developed to disseminate results of public interest prior to the conclusion of the peer-review process [38].

In such context, the use of social media (especially Twitter) played a crucial role in many cases, facilitating real time global communication among experts in an unprecedented scale [39].

The sharing of large amount of data from different sources (epidemiological data on the disease, demographic data, data coming from perception investigation, geolocation data) made it possible to have integrated data to be used for the production of scientific evidence for evidence-based prevention actions [40,41].

Another important point concerns engagement modalities. Debates among scientists brought together different opinions, especially on non-consolidated knowledge about an evolving situation. The way in which debates were carried out mirrors mass communication. The exchange of different opinions during talk shows sometimes generated intense debates [42].

Another fact worth mentioning is the need for immediate policy actions to tackle the pandemic. For this reason, many governments around the world created national committees to inform and accelerate the policy advising process. Almost all actions by the Italian government were anchored in the scientific community, with the creation of the CTS, composed of experts and qualified representatives of State Bodies and Administrations. Attention was also directed to the progress of knowledge produced by international, European and national scientific institutions [43]. The CTS was thus characterized by multidisciplinarity and cooperation between institutions, constituting a model that was later adopted in other countries. It also facilitated a fast response in the various aspects and structures of the national health system and promoted the development of technical documents, guidelines, and monitoring reports covering all areas. 

Furthermore, the CTS guaranteed technical support to the Department of Civil Protection and to the structure of the special commissioner for the COVID-19 emergency, for the acquisition of goods and services including medical equipment and personal protective equipment.

In such context, initiatives were taken for the production and dissemination of informative material aimed at providing clear and direct indications and based on scientific evidence. Such products were adapted to different work and life contexts, to promote individual and collective behaviors for the containment of risk exposure and possible infection (e.g., home isolation, COVID-19 symptoms) and to help the population understand the rules established after emergency regulations.

This type of action was not only aimed at transferring information, but also at stimulating accountability and empowerment of the citizens, by providing them with tools to facilitate their contribution to the fight against the pandemic.

The efforts to produce informative material, which was progressively updated as new evidence became available, was undertaken in a synergistic and constant manner by various public institutions generally providing scientific advice to the Government in the field of public health, national health system and occupational health, each one according to its own characteristics and specific competence. Examples are: (i) reports and ad interim indications developed by ISS, along with informative tools in several languages; (ii) good practices collected and published by the Italian National Agency for Regional Health Services (AGENAS) concerning communication, support and information to citizens, organizational solutions for COVID-19 patients, the clinical treatment of COVID-19 patients, rearrangements of clinical and assistance practice; and (iii) tutorials and publications developed by INAIL providing practical information for the management of COVID-19 risk at workplaces.

## 5. Discussion

Learning from previous experiences of infectious disease outbreak in the last decades, risk communication and community involvement have been identified as crucial elements for effective response to public health emergencies [44].

However, many things have changed, including the virus and its spreading, the way in which people gather and look for information, and the way in which the authorities communicate with the public through social media [45]. For such reasons, understanding the impact of risk communication on psychosocial and behavioral changes in large-scale epidemics and pandemics is at its inception [46]. Different models were developed for the promotion of a participatory approach for risk communication, but research on their application in different social and cultural contexts appears to be limited [47].

Furthermore, it should be noted that different aspects such as cultural, gender, educational, economical and geographical determinants, or variables such as beliefs, social norms, historical context (e.g., experience with previous epidemics), can influence risk perception, decision-making processes, and psychological and behavioral response [48].

It is essential to refine our understanding of how the public and its subgroups perceive risks, not only to improve communication strategies, but also to support public health actions during epidemics, especially considering the different players involved at different levels in the various communities.

For such reason, it is necessary to develop new and effective communication strategies aimed at facing uncertainties and challenges of risk communication in epidemics and pandemics. Such strategies should consider interaction between public health, human and social sciences, political science, law, ethics, communication and media studies [49]. 

Some authors have remarked on the lack of a coherent approach of science policy, underlining that institutions and the scientific community are not sufficiently aware of their roles, and of the importance of a transparent and effective interface between science and policy that can inspire confidence throughout the public and promote correct behaviors for infection prevention [50].

The idea is to favor a continuous process of risk communication, bidirectional and dialogic, that could allow for the widest possible dissemination of information and opinions among institutions, scientific community, social media and public, following the traditional principles of public communication: clarity, transparency, promptness, identifiability, consistency, and the reliability of information [51,52].

In a context where scientific knowledge about the novel coronavirus has been evolving continuously and rapidly, we consider it suitable for communication to follow the same steps of preparation, monitoring, intervention, and reinforcement that characterize pandemic plans, making communication a pillar.

The ways in which crisis are managed, including communication strategies, are a matter of considerable debate, especially during the phases of emergency planning.

When crises occur, the public has the fundamental right to be informed about what happens, what the potential risks are, how authorities intend to face the crisis, and what the possible consequences are. To cope with crises, everything has to be planned and prepared, including communication, which should be designed, prepared and improved to allow for fast and coordinated action. 

It is thus necessary to carry out preparedness actions that include: the adaptation of communication strategies according to different epidemiological scenarios; fast and precise information gathering on new tools for diagnosis and prevention; strategy adaptation and the improvement of social media channels, etc. [53,54].

Such principles, along with partnership among institutions and between institutions and civil society entities, are deemed indispensable for guaranteeing univocal communication in emergencies [51].

Besides communication modalities, the characteristics of the target should also be taken into account, along with needs, objectives, interests, functions of the target, bounds and arguments that may interest it.

While the scientific community played an important role in producing evidence-based knowledge and, consequently, in providing technical and scientific advice to the policymaker and guiding mass communication, a reflection would be necessary, from an ethics point of view, regarding those people who get involved in mass communication by putting in play their opinions with the risk of increasing inequity, stigma, ageism and the delay of medical care [55].

Additionally, it is crucial to specifically analyze how citizens relate to social media. As we know, mass communication has always played a key role in public response to health emergencies contributing to health awareness and promotion by changing public attitudes and influencing health behavior. This effectiveness is due to the use of a combination of different communication strategies—verbal, written and visual—as well as the function of mass communication to serve as a “bridge” that facilitates communication between governments, health institutions and the people.

However, the same characteristics of mass communication (direct, fast, interactive, etc.) aimed at a large and heterogeneous public, requires particular attention in verifying the truthfulness of the messages conveyed in order to avoid the spreading of inaccurate and altered information (infodemic). In this regard, it is necessary to adopt rapid, coordinated and systematic action by the institutional, scientific and mass communication communities in order to identify shared measures capable of protecting from any future infodemic risk. It would be useful to involve all stakeholders, including people responsible for communication strategies and policies, the media and social media platforms, researchers, technologists, civil society leaders and influencers, to further strengthen their actions to disseminate accurate information and prevent the spread of mis-and disinformation [56,57,58,59].

Those who have an important role in communication should always take into account the significance and impact of what is communicated.

## 6. Conclusions

The global COVID-19 pandemic, with all the victims, its impact on health systems and on economies, represented an unexpected and complex emergency, not only for pub-lic health, but also for communication systems. In fact, the unprecedented availability of sources of information paired with diverse and easy opportunities to access, which mark the contemporary communicative context, resulted in an overload of information, not al-ways easy to understand and manage by the public. 

The analysis of the impact of COVID-19 on risk communication in Italy that we pre-sent in this study may contribute to stimulating the discussion on the importance of communication in public health crises, and consequently to identifying practical recommendations for the improvement of current risk communication strategies. 

According to our findings, actions required to address needs and challenges for risk communication in times of pandemic must be local, and consistent with the specific characteristics of the national context, even if the same issues can be found at global level. Primarily, the need for a more inclusive risk communication, taking into account all the actors involved in the communication process (message senders and recipients), as well as different contexts in which the communication may take place. In addition, and with the specific aim of combating the infodemic [60,61], there is a clear need for greater control over the truthfulness of the information delivered, and of better coordination between institutional, scientific and mass communication. 

To this end, the integrated approach to risk communication we have drawn, which considers interconnections between different levels of communication (institutional, scientific and mass communication), tries to identify strengths (transparency, integrity, interaction, evidence-base, the ease of understanding, etc.) and weaknesses (the lack of filters for some sources, privacy and data protection, etc.), as well as opportunities (the ease of access, large audience thanks to different communication types and methods, etc.) and the threats (inadequate digital literacy, information overload; the spreading of inaccurate and altered information, etc.) of risk communication in times of pandemic. In our view, these are the starting points for strategic actions able to ensure a more efficient risk communication. 

## Data Availability

Not applicable.
